# Peptidases from Latex of *Carica candamarcensis* Upregulate COX-2 and IL-1 mRNA Transcripts against *Salmonella enterica* ser. Typhimurium-Mediated Inflammation

**DOI:** 10.1155/2014/819731

**Published:** 2014-03-13

**Authors:** Maria Taciana Ralph, Ayrles Fernanda Brandão Silva, Dayane Laíse da Silva, Danielle Cristina Oliveira do Nascimento, Diogo Manoel Farias da Silva, Manoel A. Gomes-Filho, Paulo Roberto Eleutério Souza, Joaquim Evêncio-Neto, Márcio Viana Ramos, Carlos Edmundo Salas, José Vitor Lima-Filho

**Affiliations:** ^1^Departamento de Biologia, Universidade Federal Rural de Pernambuco, 52171-900 Recife, PE, Brazil; ^2^Departamento de Bioquímica e Biologia Molecular, Universidade Federal do Ceará, 60020-181 Fortaleza, CE, Brazil; ^3^Departamento de Morfologia e Fisiologia Animal, Universidade Federal Rural de Pernambuco, Recife, PE, Brazil; ^4^Departamento de Biologia, Facultad de Ciencia, Universidade de La Serena, La Serena, Chile; ^5^Rua Dom Manoel de Medeiros, s/n, Departamento de Biologia, Laboratório de Microbiologia e Imunologia (LAMIM), Universidade Federal Rural de Pernambuco, Campus Dois Irmãos, CEP, 52171-900 Recife, PE, Brazil

## Abstract

The immunomodulatory properties of a mixture of cysteine peptidases (P1G10) obtained from the fruit lattice of *Carica candamarcensis* were investigated. P1G10 was obtained from fresh latex samples by chromatography in a Sephadex column and initially administered to Swiss mice (*n* = 5; 1 or 10 mg/kg) via i.p. After 30 min, the mice were injected with carrageenan (0.5 mg/mouse) or heat-killed *S.* Typhimurium (10^7^ CFU/mL; 100°C/30 min) into the peritoneal cavity. Afterwards, two animal groups were i.p. administered with P1G10 (*n* = 6; 1, 5, or 10 mg/Kg) or PBS 24 hours prior to challenge with live *S.* Typhimurium (10^7^ CFU/mL). P1G10 stimulated the proliferation of circulating neutrophils and lymphocytes, 6 h after injection of carrageenan or heat-killed bacteria, respectively. Furthermore, survival after infection was dose-dependent and reached 60% of the animal group. On the other hand, control mice died 1–3 days after infection. The examination of mRNA transcripts in liver cells 24 h after infection confirmed fold variation increases of 5.8 and 4.8 times on average for IL-1 and COX-2, respectively, in P1G10 pretreated mice but not for TNF-**α**, IL-10, **γ**-IFN and iNOS, for which the results were comparable to untreated animals. These data are discussed in light of previous reports.

## 1. Introduction

*Carica candamarcensis* (Caricaceae) is a plant native to western South America, popularly known as mountain papaya. After injury, the leaves and outer layers of the endocarp release a large amount of latex that is rich in carbohydrates, vitamins, minerals, and proteins [1, 2]. Previously, laticifer protein fractions from* C. candamarcensis* were obtained by chromatography in a Sephadex column named P1G10 and P2G10 [3]. The P1G10 fraction is rich in cysteine peptidases with proteolytic activity at least five times higher than* C. papaya* [1, 4].

Plant peptidases are usually implicated in protein processing in response to different external stimuli and in removal of misfolded proteins [5]. These properties have proven beneficial for the treatment* in vivo* of worm infections caused by* Heligmosomoides polygyrus* with papaya latex, since peptidases promote digestion of the worms' cuticle [6]. In particular, the fraction P1G10 of* C. candamarcensis* has also shown antiulcerogenic activity, similar to omeprazole or ranitidine, with effects on gastric healing that were correlated with an angiogenic stimulus [7]. P1G10 has mitogenic properties in cultured fibroblasts and epithelial cells [3] related to two peptidases already identified: CMS2MS2 and CMS2MS3 [8]. Moreover, shortening of epithelialization in a skin burn injury model was shown when P1G10 was applied at a concentration of 0.1% [9].

Previously we have shown that plant laticifer proteins can potentially be used for control of inflammatory processes derived from systemic infections [10]. Using a model of typhoid fever in rodents caused by* S. enterica* ser. Typhimurium, we have shown that cysteine peptidases from the laticifer fluid of* Calotropis procera* influence the release of proinflammatory cytokines, exhibit thrombin- and plasmin-like activities, and maintain coagulation homeostasis in septic mice [11, 12]. These protein fractions have been exploited in immunotherapy, since plant peptidases can potentially mimic endogenous human peptidases involved, for example, with coagulation, digestion, and apoptosis. In the present study, the role of P1G10 of* C. candamarcensis* in inflammatory response was investigated in Swiss mice experimentally infected with* S.* Typhimurium.

## 2. Methods

### 2.1. *C. candamarcensis* Latex Proteins

A voucher specimen of the plant was deposited at the herbarium of the Universidad de La Serena, Chile (number 15063). The latex was collected by several incisions on the surface of the green fruit with sharp blade. After collection, it was lyophilized and stored in the dark at −20°C until use. Then the sample was dissolved and equilibrated with sodium acetate buffer 1 M (pH 5.0) and applied in a Sephadex G10 column. The C18 column (Vydac) was equilibrated with 27% acetonitrile in 0.1% trifluoroacetic acid (TFA) before injection of 1 mg P1G10 dissolved in milli-Q water, followed by elution with a nonlinear acetonitrile-0.1% TFA gradient. Protein measurement was performed using egg-white lysozyme as standard, according to Lowry et al. [13]. The samples were screened by determining the absorbance at 280 nm and the amidase activity [3]. The first peak with amidase activity constituted the fraction P1G10.

### 2.2. Microorganisms

*Salmonella enterica* subsp.* enterica* ser. Typhimurium were isolated from a human clinical case sample, maintained by Ezequiel Dias Foundation (FUNED, Belo Horizonte, MG, Brazil) and was kindly provided by Dr. Jacques Robert Nicoli (Federal University of Minas Gerais, Brazil). The bacteria were kept at −18°C in brain heart infusion (BHI) culture medium containing 50% glycerol. During the experiments, the bacteria were activated by being cultured in BHI broth for 24 hours at 37°C.

### 2.3. *In Vitro* Antimicrobial Activity

The antimicrobial activity of P1G10 was assessed using the broth dilution method [14]. The proteins were dissolved in phosphate buffered saline (PBS) to give final concentrations ranging from 1 mg/mL to 0.0019 mg/mL in Mueller-Hinton broth tubes containing* S.* Typhimurium (10^5^ CFU/mL). All assays were performed in duplicate. Control tubes lacked latex proteins. The minimum inhibitory concentration was estimated as the lowest concentration that inhibited visible growth after incubation for 24 h at 37°C.

### 2.4. Animals

All procedures were conducted in accordance with internationally accepted principles on the use of laboratory animals and were approved by the experimental ethics committee of Pernambuco Federal Rural University (Process 23082.012528/2012). Swiss mice (*Mus musculus*) were obtained from the collection of the Keizo Asami Immunopathology Laboratory (LIKA) of Federal University of Pernambuco. The mice weighed 30–35 grams and were kept in cages with controlled lighting (12 h light/dark cycles), 25°C with free access to water and commercial feed (Purina, Paulínia, SP, Brazil). The experiments were performed at the Microbiology and Immunology Laboratory of Federal Rural University of Pernambuco.

### 2.5. Experimental Peritonitis Induced by Carrageenan or Heat-Killed* S.* Typhimurium

The protocol was adapted from Alencar et al. [15]. The protein fraction P1G10 of* C. candamarcensis *was administered at concentrations of 1 or 10 mg/kg intraperitoneally (i.p.) to uninfected Swiss mice (*n* = 5). The control groups were given PBS. After 30 minutes; the peritonitis was induced by i.p. administration of 0.2 mL of a carrageenan solution (0.5 mg/animal) or heat-killed* S.* Typhimurium (10^7^ CFU/mL; 100°C/30 min). After 6 h, the animals were euthanized under anesthesia with isoflurane. For the total leukocyte count in the blood and peritoneal fluid, 20 *μ*L was homogenized with 380 *μ*L of the Turk reagent. An aliquot of this solution was withdrawn and placed in a Neubauer chamber, and leukocytes were counted under an optical microscope. The differential count was made from smears stained with eosin methylene blue-Giemsa [16].

### 2.6. Experimental Infection with* S.* Typhimurium

The protocol was adapted from Lima-Filho et al. [10]. Mice were divided into four groups (*n* = 6) as follows: experimental groups were i.p. administered with 0.2 mL of P1G10 in sterile PBS (137 mM NaCl, 10 mM Na_2_HPO_4 _2H_2_O, 2 mM KH_2_PO_4_), pH 7.2, at quantities of 1, 5, or 10 mg/kg, while the control group received 0.2 mL of PBS. After 24 hours, all animals were challenged also by the i.p. route with 0.2 mL of a suspension containing 10^7^ CFU/mL of* S.* Typhimurium. The clinical symptoms and survival after infection were monitored every 24 hours for seven days. Surviving animals were subjected to euthanasia with isoflurane at the end of experiment. Two other animal groups (*n* = 10 each), administered with P1G10 (10 mg/kg) or PBS and infected with* S.* Typhimurium, were submitted to euthanasia 24 h or 72 h after infection, and samples of blood, peritoneal fluid, spleen, and the liver were collected for analysis.

### 2.7. Assessment of the Bacterial Clearance after Infection

The spleen and livers were removed aseptically and homogenized in PBS pH 7.2. The suspensions of these organs and samples of blood were submitted to serial decimal dilutions. Then, an aliquot of 0.1 mL of each dilution was plated on a MacConkey agar plate. The plates were incubated in a growth chamber for 24 h at 37°C for later quantification of the colony forming units [11].

### 2.8. Histopathological Analysis

Tissue samples of spleen and liver were collected after 72 h of infection of all animal groups, fixed in 10% formaldehyde and embedded in paraffin blocks. Histological sections with 5 *μ*m thickness were stained with hematoxylin-eosin. The slides were examined by a single pathologist, who was unaware of the experimental conditions.

### 2.9. Assessment of Gene Expression of Inflammatory Mediators

A section of each animal's liver was removed aseptically and homogenized in 0.5 mL of TRIzol Reagent (Invitrogen) to extract total RNA, following the protocol suggested by the manufacturer. Quantification of RNA was performed by spectrophotometry at 260 nm and its integrity was confirmed by electrophoresis on 1% agarose gel [11]. The construction of cDNA and amplification by real-time PCR were performed using a commercial kit, following the manufacturer's instructions (Sigma-SYBR-Green Quantitative RT-PCR kit).

The following primers were used: *β*-actin Mouse (Internal Control) ATATCGCTGCGCTGGTCGTC 3′, 5′ AGGATGGCGTGAGGGAGAGC 3′; tumor necrosis factor-*α* (TNF-*α*) (5′ GATCTCAAAGACAACCAACTAGTG3′, 5′ CTCCAGCTGGAAGACTCCTCCCAG 3′); Ciclooxigenase-2 (COX-2) (5′ AGTTTTT CAAGACAGATCATAAGCG 3′, 5′ TGCTCCTGCTTGAGATGTCG 3′); inducible Nitric Oxide Synthase (iNOS) (5′ AAGCACATGCAGAATGAGTACCG 3′, 5′ GTGGGAC AGCTTCTGGTCGAT 3′); interleukin-10 (IL-10) (forward 5′ CGGGAAGACAATAACTG 3′, reverse 5′ CATTTCCGATAAGGCTTG 3′); and gamma-interferon (*γ*-IFN) (5′ GGTGAC ATGAAAATCCTGCAGAGC 3′, 5′ CGCTGGACCTGTGGGTTGTTGACC 3′).

The results were analyzed according to Dussault and Pouliot [17]. For comparison of the levels of expression of genes of interest (G.I.) between control and experimental groups, we used the following formula:

(1) ΔΔCt=[(Ct  G.I.  Control−Ct  Actin  Control)  −(Ct  G.I.  Experimental     −Ct  Actin  Experimental)].

The results were expressed in terms of fold variation with formula: 2^ΔΔCT^.

### 2.10. Quantification of Nitric Oxide in Serum of Animals

The determination of nitric oxide in blood serum was carried out in the form of nitrite. NO^•^ was first transformed into NO_3_^−^ by dioxygenation. The conversion of nitrate (NO_3_^−^) into nitrite (NO_2_^−^) was performed by the action of the enzyme nitrate reductase, using a commercial kit (R&D Systems).

### 2.11. Statistical Analysis

The results are expressed as mean ± standard error of the mean (SEM). For comparison of multiple parametric data, we used analysis of variance (ANOVA) followed by Bonferroni's test or the Student-Newman-Keuls test. The significance level was *P* < 0.05. The analysis and processing of the results were performed using the GraphPad Prism program (version 5.0).

## 3. Results 

P1G10 was not bacteriostatic or bactericidal against the strain of* S.* Typhimurium used for all experiments described here at any of the dosages tested. Pretreatments with P1G10 did not significantly increase the infiltration of leukocytes into the peritoneal cavity induced by carrageenan or heat-killed* Salmonella *(*P* > 0.05) (data not shown). However, the number of neutrophils in the bloodstreams of P1G10-pretreated mice was increased after injection with carrageenan (*P* < 0.05), but not eosinophils, basophils, or lymphocytes (Table 1). Moreover, pretreatments with P1G10 increased the number of circulating lymphocytes when mice were injected with heat-killed* Salmonella *(*P* < 0.05) (Table 1).

**Figure 1:**
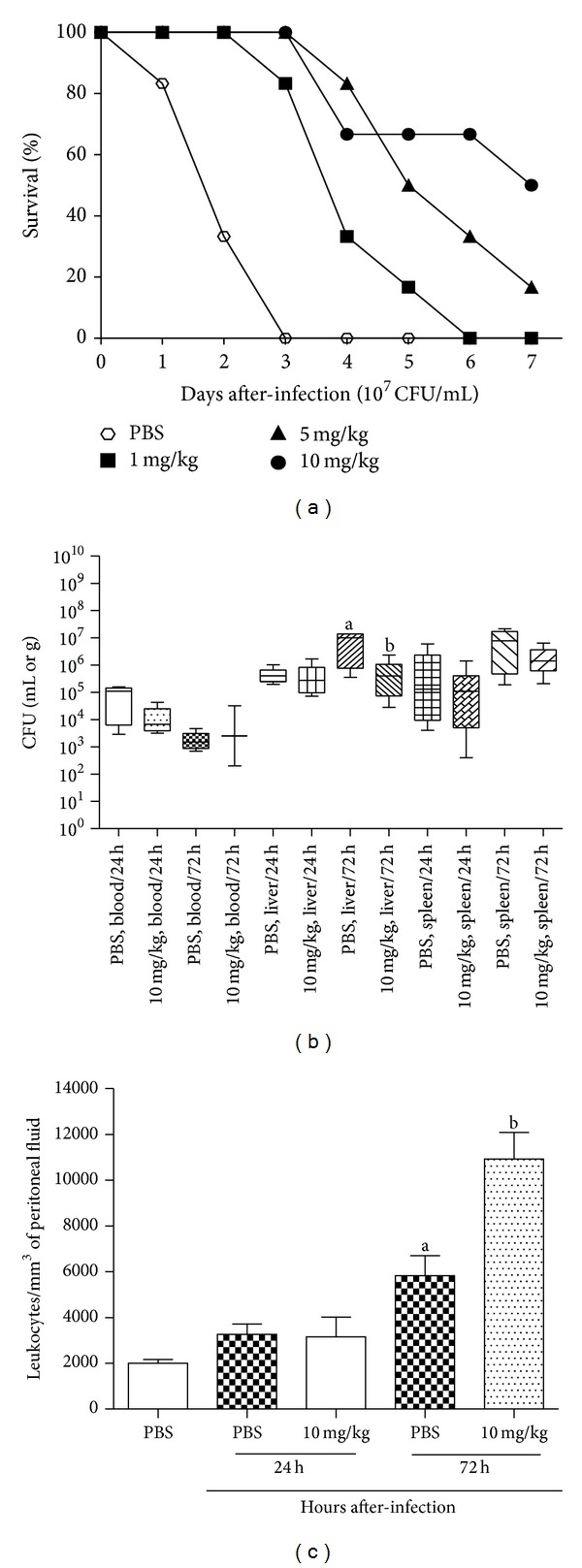
Survival, bacterial clearance and inflammation in Swiss mice pretreated with P1G10 and infected with* Salmonella* Typhimurium. Survival was evaluated in mice pretreated with 1, 5, or 10 mg/kg of PIG10 intraperitoneally (a). The control groups received PBS (b). The bacterial cell counts in blood, spleen, and the liver were evaluated 24 h and 72 h after infection as well as the total leukocyte cell counts in the peritoneal fluid (c). Results are expressed as mean ± SD and data comparison was by Student's *t*-test or analysis of variance (ANOVA) followed by the Turkey test. The confidence interval was determined with *P* < 0.05. Different superscript letters mean significant difference between P1G10 and PBS groups.

Survival of Swiss mice pretreated with P1G10 was dose-dependent and reached 60% of the animal group after seven days' infection with* Salmonella *(*P* < 0.05) (Figure 1(a)). After 24 and 72 h of infection, the number of viable bacteria in blood and the spleen did not differ significantly between the animal groups (*P* > 0.05). But the bacterial load in the liver of mice pretreated with P1G10 was significantly reduced 72 h after infection (*P* < 0.05) (Figure 1(b)). Conversely, untreated animals (PBS group) died 1–3 days after infection. There was a high infiltration of leukocytes into the peritoneal cavity of P1G10-pretreated mice 72 h after infection (*P* < 0.05) (Figure 1(c)). But the total leukocyte counts in the bloodstreams or the hematological profile of leukocyte populations was similar between the animal groups (data not show).

**Figure 2:**
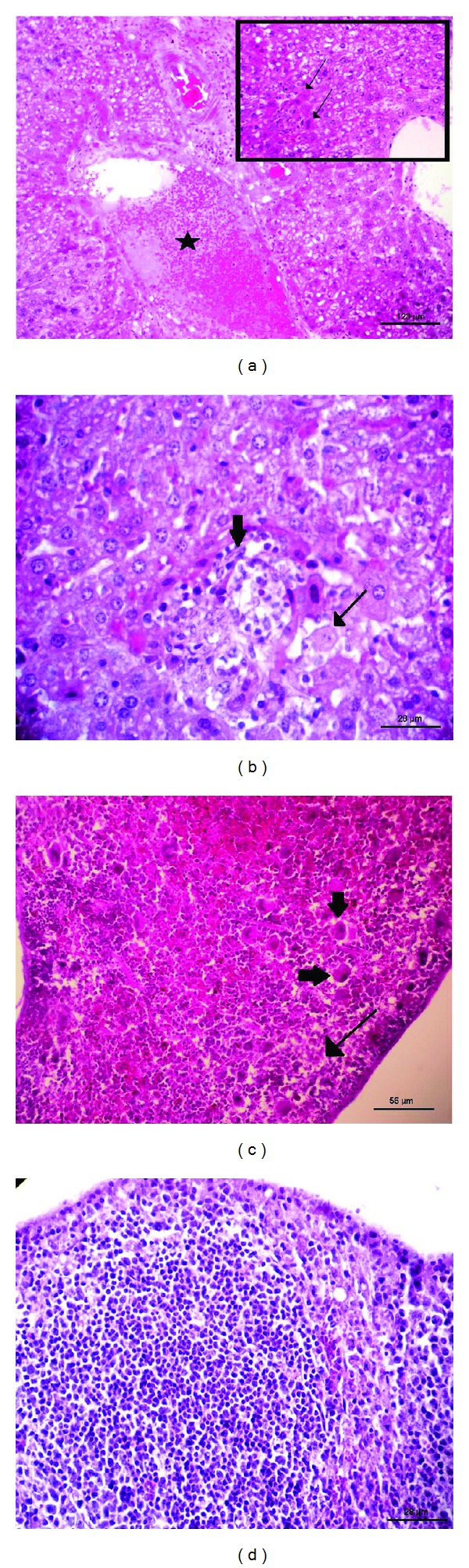
Histologic pattern of the liver and spleen of mice pretreated with P1G10 72 hours after infection by* Salmonella* Typhimurium. (a) Liver of mice in the PBS group, showing severe vacuolation, necrosis of individual hepatocytes with diffuse distribution (thin arrow), and thrombosis (star); (b) liver of mice in the experimental group (P1G10, 10 mg/Kg), demonstrating hepatic necrosis (thin arrow) and accumulation of mononuclear cells (large arrow); (c) spleens of mice in the control group, showing lymphoid depletion (thin arrow) and the presence of giant cells (large arrow); (d) spleens of mice in the experimental group with preserved appearance of the white pulp and red pulp.

The histological evaluation of the liver of the control animals after 72 h of infection revealed inflammatory infiltration, thrombus formation, pyknotic nuclei, hepatic necrosis, and intense diffuse cytoplasmic vacuolization (Figure 2(a)). In the same period, the liver of animals pretreated with P1G10 showed mild vacuolization of hepatocytes, mild signs of necrosis, presence of microabscesses and accumulations of mononuclear cells (Figure 2(b)). The analysis of the spleen of control animals 72 h after infection revealed that the presence of splenic parenchyma in areas of inflammation and necrosis in lymphoid depletion with multinucleated cells (Figure 2(c)), while the spleen of animals pretreated with P1G10 only had slight inflammation and absence of multinucleated giant cells (Figure 2(d)).

**Figure 3:**
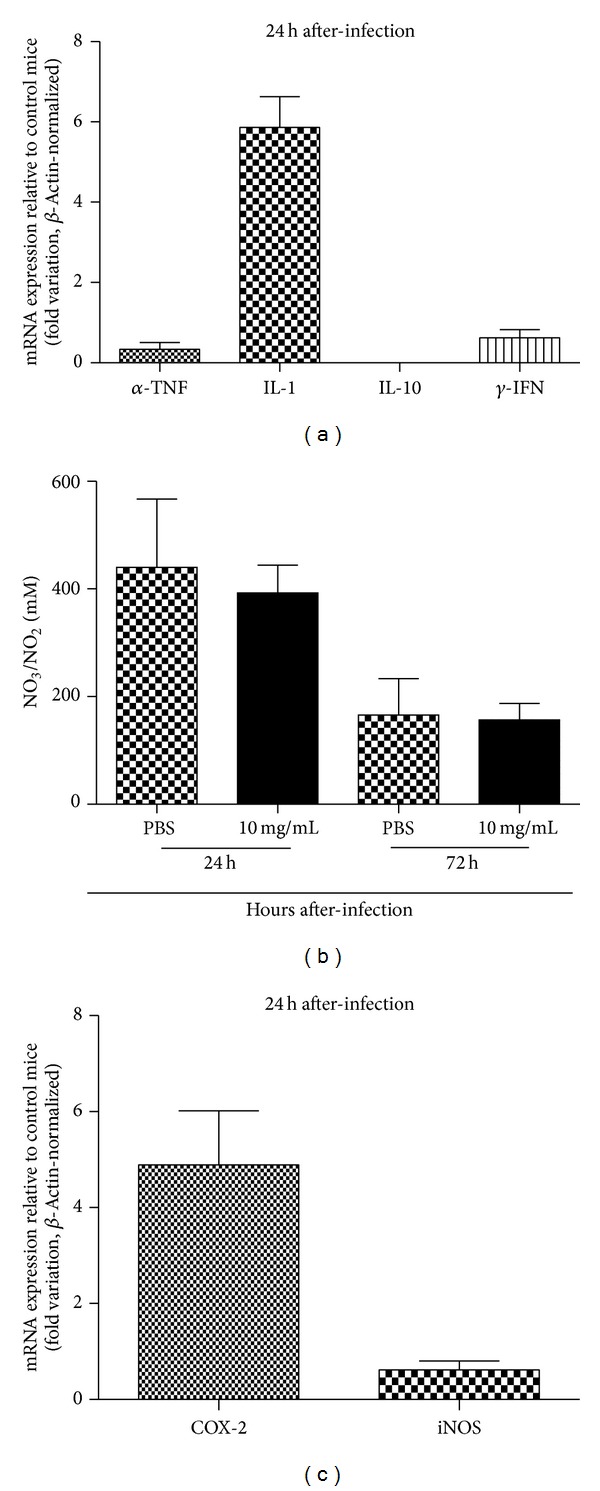
mRNA expression of cytokines and inducible COX-2 and iNOS enzymes besides nitric oxide in serum of P1G10 pretreated mice infected with* Salmonella* Typhimurium. Comparative real-time PCR results are expressed in fold variation relative to control mice (a and b). Beta-actin was used as internal control. Nitric oxide release in serum was titrated in nitrite form (c). In this case, data comparison was conducted with Student's *t*-test (b). The confidence interval was determined with *P* < 0.05.

The examination of mRNA transcripts of inflammatory mediators in liver cells 24 h after infection confirmed fold variation increases of 5.8 and 4.8 on average for IL-1 and COX-2, respectively, in mice pretreated with P1G10 (Figures 3(a) and 3(b)). Conversely, the gene expression levels of TNF-*α*, IL-10, *γ*-IFN, and iNOS were comparable to untreated animals (Figure 3(a)). Indeed, the NO^•^ serum levels in experimental mice were not particularly altered in comparison to the control ones 72 h after infection (Figure 3(c)).

## 4. Discussion

*S.* Typhimurium provokes a syndrome similar to the human typhoid fever caused by* S.* Typhi in the murine model, which has been used to study severe inflammatory processes derived from bacterial infections [18, 19]. Systemic infections caused by* Salmonella* typically induce the failure of leukocyte migration to the infection site, among other inflammatory disorders, leading to septic shock and death [20, 21]. In the present study, we have shown that P1G10 has immunomodulatory properties, which varied between infected and uninfected models.

Preliminary assays that used carrageenan or heat-killed* Salmonella* as phlogistic agents have shown that P1G10 stimulated the proliferation of circulating neutrophils and lymphocytes, which are important for resistance against salmonelosis [22]. Although the hematological profile was not particularly affected in a real infection, pretreatments with P1G10 increased the survival of mice after a lethal inoculum of* S.* Typhimurium in a dose-dependent manner. This protective effect was confirmed through histological examination in the main target organs of infection, the liver and spleen. Additionally, the infiltration of leukocytes into the peritoneal cavity was only significant in mice pretreated with P1G10 after infection (but not after carrageenan or heat-killed* Salmonella* injection). Since* Salmonella* actually have the ability to grow in the peritoneal cavity after i.p. inoculums, we hypothesize that P1G10 immunomodulation was boosted by the continuous intracellular bacterial growth into target organs during the infection.

As an attempt to explain the above results, we investigated the influence of P1G10 on inflammatory mediators. The activation of proinflammatory cytokines such as TNF-*α* and IL-1*β* is important for host resistance against* Salmonella*, since they induce maturation of dendritic cells with implications on initiation of the adaptive immune response [23]. Moreover, nitric oxide (NO^•^) is often released after the inducible nitric oxide synthase (iNOS) is triggered by cytokines such as TNF-*α* and IFN-*γ* [19]. However, our data show that mRNA expression levels of both TNF-*α* and IFN-*γ* were comparable to the control mice, which died one to three days after infection. In spite of the microbicidal role of NO^•^ in the intracellular environment, which could explain the lower number of colony forming units in the liver of experimental mice 72 h after infection, it was apparently not decisive to increase survival of experimental mice.

High serum levels of TNF-*α* have been shown to increase the severity of septic infections, while mice deficient in the production of IL-1*β* are resistant to septic shock [24–26]. On the other hand, mRNA transcripts of IL-1*β* and COX-2 were upregulated in mice pretreated with P1G10 after infection. Interleukin-1*β* has as main source macrophages stimulated by TNF-*α* or bacterial LPS and is responsible for promoting the recruitment of leukocytes to the infection site [20]. Furthermore, COX-2 enzyme is inducible by inflammation, converting arachidonic acid to prostaglandin, with its regulation being dependent on IL-1 among other stimuli [27]. Prostaglandin E_2_ (PGE_2_) exerts local inflammation and phagocyte-mediated immunity at the infection site but has anti-inflammatory properties in extended immune responses [28]. Although* Salmonella* infection naturally augments COX-2 mRNA expression and PGE_2_ synthesis [29], P1G10 pretreatments apparently contribute to recruitment and activation of leukocytes in early infection.

Previous studies have shown different effects of plant peptidases on inflammatory response. For example, bromelain, a mix of peptidases derived from pineapple stem with anti-inflammatory properties, affects T-cell mRNA expression of IL-2, IL-4, and IFN-*γ* in colon epithelial cell lines [30, 31]. Bromelain also blocks* S.* Typhimurium induced ERK-1, ERK-2, and c-Jun NH2-terminal kinase (JNK) activation in Caco-2 cells [32]. Additionally we have shown that laticifer peptidases of* C. procera* promote downregulation of IL-1*β*, among other inflammatory mediators, during infection by* S.* Typhimurium [10, 11]. In general, plant peptidases appears to exert their biological actions indirectly on immune cells due to the proteolytic cleavage of membrane surface molecules triggering (or abrogating) the inflammatory cascade [33, 34]. For instance, peptidases of* C. candamarcensis* is shown to stimulate proliferation of specific cell lines such as L929, MDA-MB231, and BHK-21 [35], and this effect was further associated with an increase in activity of Erk2, a component of the MAP kinase pathway [8]. Since the MAPK/ERK pathway is involved in IL-1*β*-induced COX-2 expression and PGE_2_ production [36], it is reasonable to assume that pretreatments with P1G10 potentially influenced the MAP kinase pathway* in vivo* after infection by* Salmonella*.

Although the specific mechanism enhanced by P1G10 in the immune system is still to be elucidated, we have shown that early activation of inducible proinflammatory mediators possibly contributed to local bacterial killing, increasing the survival of mice after infection. Moreover, activation was not downregulated by IL-10, since no mRNA transcripts were detected for the cytokine in early infection. Since medicinal plants have been traditionally used for prevention of infectious diseases caused by different etiological agents, the clinical relevance of P1G10 is clear. Finally, we conclude that P1G10 is a source of immunomodulatory proteases that could be used for activation of the immune response against intracellular pathogens.

## Figures and Tables

**Table 1:** Leukocyte cell counts in the bloodstream of Swiss mice pretreated with P1G10 6 h after injection of carrageenan or heat-killed *S.* Typhimurium into the peritoneal cavity.

	Total leukocytes (10^3^ cells/mm^3^)	Differential leukocyte cell counts (10^3^ cells/mm^3^)				
		Eosinophil	Lymphocytes	Neutrophils	Monocytes	Basophils
P1G10 (i.p) + 0.5 mg carrageenan (i.p.)						
PBS	5.90 ± 1.30	0.0 ± 0.0	3.58 ± 1.10	2.23 ± 0.53	0.09 ± 0.04	0.0 ± 0.0
1 mg/Kg	4.37 ± 2.25	0.0 ± 0.0	2.69 ± 1.31	1.64 ± 0.97	0.04 ± 0.03	0.0 ± 0.0
10 mg/kg	8.52 ± 1.56*	0.0 ± 0.0	4.44 ± 1.22	4.02 ± 0.45*	0.06 ± 0.04	0.0 ± 0.0
P1G10 (i.p.) + Heat-killed *Salmonella* (i.p.)						
PBS	4.40 ± 0.98	0.0 ± 0.0	2.93 ± 0.68	1.41 ± 0.30	0.05 ± 0.04	0.0 ± 0.0
1 mg/Kg	4.32 ± 1.54	0.0 ± 0.0	3.10 ± 1.07	1.17 ± 0.48	0.05 ± 0.03	0.0 ± 0.0
10 mg/kg	6.72 ± 0.66*	0.0 ± 0.0	4.63 ± 0.65*	2.01 ± 0.59	0.08 ± 0.06	0.0 ± 0.0

*Significant difference in comparison to PBS group (*P* < 0.05).
